# Down-Regulation of Desmosomes in Cultured Cells: The Roles of PKC, Microtubules and Lysosomal/Proteasomal Degradation

**DOI:** 10.1371/journal.pone.0108570

**Published:** 2014-10-07

**Authors:** Selina McHarg, Gemma Hopkins, Lusiana Lim, David Garrod

**Affiliations:** Faculty of Life Sciences, University of Manchester, Manchester, United Kingdom; University of Colorado, Boulder, United States of America

## Abstract

Desmosomes are intercellular adhesive junctions of major importance for tissue integrity. To allow cell motility and migration they are down-regulated in epidermal wound healing. Electron microscopy indicates that whole desmosomes are internalised by cells in tissues, but the mechanism of down-regulation is unclear. In this paper we provide an overview of the internalisation of half-desmosomes by cultured cells induced by calcium chelation. Our results show that: (i) half desmosome internalisation is dependent on conventional PKC isoforms; (ii) microtubules transport internalised half desmosomes to the region of the centrosome by a kinesin-dependent mechanism; (iii) desmosomal proteins remain colocalised after internalisation and are not recycled to the cell surface; (iv) internalised desmosomes are degraded by the combined action of lysosomes and proteasomes. We also confirm that half desmosome internalisation is dependent upon the actin cytoskeleton. These results suggest that half desmosomes are not disassembled and recycled during or after internalisation but instead are transported to the centrosomal region where they are degraded. These findings may have significance for the down-regulation of desmosomes in wounds.

## Background

The intercellular adhesive strength of the epidermis and myocardium enables these tissues to withstand mechanical stress. Desmosomes are intercellular junctions that mediate this strong adhesion. By joining adjacent cells and binding to the keratin intermediate filament (IF) network, desmosomes act as linkers providing adhesion and great tensile strength. The importance of desmosomes is highlighted by the severe skin and cardiac defects that arise in autoimmune and genetic diseases [1]–[5].

Desmosomes are complex, transversely symmetrical structures composed of five main proteins. The desmosomal cadherins, desmoglein (Dsg) and desmocollin (Dsc), form the adhesive interface of the desmosome and their cytoplasmic tails bind to the armadillo proteins, plakoglobin (PG) and plakophilin (PKP), in the desmosomal plaque. The armadillo proteins in turn bind to desmoplakin (DP), which links the desmosome to the IFs [6]–[10].

Strong adhesion, though essential for tissue integrity, is incompatible with tissue remodelling such as takes place during epidermal wound healing and embryonic development. To facilitate remodelling, adhesion must be down-regulated but the mechanisms which govern down-regulation of desmosomes remain poorly understood. Ultrastructural studies of wound edge epidermis clearly show that entire desmosomes are internalised by cells [11], [12]. Once internalised, they are presumably degraded. Alternatively, they may be internally disassembled and their component proteins recycled.

In the context of tissue remodelling we have shown that desmosomes, both in culture and in vivo, can adopt two alternative adhesive states [12]–[15]. In normal tissues and confluent monolayers, desmosomes adopt calcium-independent adhesion, termed hyper-adhesion [12]–[13], [15]–[16]. However, in subconfluent epithelial cultures [15], early embryogenesis and wound re-epithelialisation [12], [14], [16] desmosomal adhesion becomes calcium dependent. Hyper-adhesive desmosomes are more strongly adhesive than calcium dependent desmosomes [13]. The switch from hyper-adhesion to calcium dependence appears to be triggered by cell signalling since it (a) occurs without any qualitative or quantitative change in the major desmosomal components and (b) is triggered by activation of protein kinase C (PKC) or inhibition of protein phosphatases [13], [15]. Moreover, the knockdown or knockout of PKCα promotes desmosomal hyper-adhesion [15]–[16].

On chelation of extracellular calcium, calcium dependent desmosomes have been shown by electron microscopy to split into half desmosomes that are rapidly internalised by the cells [17]. Calcium switching is widely regarded as an accepted method to study the assembly of desmosomes in tissue culture, and also, but perhaps less commonly, to study desmosome breakdown [17]–[20]. While calcium switching is unphysiological, in terms of desmosome breakdown it has the merit that it involves the vacuolar internalisation of complex structures, half desmosomes, and thus, to some extent reassembles the process that has been described in vivo. Half desmosomes are also produced by trypsinisation [21] and so is a daily occurrence when epithelia cells are passaged in culture. We have therefore used this model in order to attempt to provide novel information that may be relevant to the down-regulation of whole desmosomes.

We postulated that PKC signalling somehow primes the desmosomes in wounds for internalisation [12]. In the present study we test the role of PKC, and investigate both the role of the cytoskeleton in internalisation and internal transport and the fate of internalised desmosomal halves. Our results support a role for PKC and actin in internalisation. Once internalised, half desmosomes are transported to the centrosomal region by microtubules. Furthermore, internalised half desmosomes are not disassembled or recycled but are degraded by the combined action of lysosomes and the proteasome.

## Materials and Methods

### Cell culture

HaCaT cells [22] (a gift from Dr N.Fusenig), Madin Darby canine kidney type II cells **(**MDCK) [23] (ECACC, UK) and MDCK cells stably expressing Dsc2a-YFP (a gift from R.E.Leube) [24] were cultured in standard normal calcium medium (1.7 mM CaCl_2_) (NCM) consisting of Dulbecco's Modified Eagle's Medium (DMEM) supplemented with 10% Foetal Calf Serum (FCS) (Sigma, Poole, UK) and 100 U/ml penicillin and 100 µg/ml streptomycin at 37°C in 5% humidified CO_2_.

### Low calcium medium and drug treatment of cells

Cells were seeded at a subconfluent density of 70,000 cells/cm^2^ in NCM on 13 mm diameter coverslips in 24 well plates for 24 hours. They were then washed 3 times in calcium and magnesium free HBSS (CMF HBSS) and incubated in calcium free DMEM (Life Technologies, Paisley, UK) supplemented with 10% chelated FCS and 3 mM EGTA (LCM) for 60 minutes (unless specified otherwise) at 37°C in 5% humidified CO_2_. To assess the role of actin, microtubules and conventional PKC isoforms in desmosome internalisation, MDCKs were pre-incubated with 5 µM latrunculin A, 33 µM nocodazole or 0.8 µM Gö6976 (all from Sigma) for 20–30 minutes. MDCK cells were then co-treated with LCM and the relevant inhibitor or vehicle (DMSO) alone for 60 minutes. To assess the role of kinesins in the internal transport of desmosomes, MDCK cells were treated in an identical manner with 500 µM adenylyl-imidodiphosphate (AMP-PNP) or 50 µM aurintricarboxylic acid (ATA) (Sigma) or vehicle alone (0.1% ethanol). Cells were then fixed in methanol and stained for immunoflourescence.

To investigate the degradation of desmosomal proteins cells were seeded at a subconfluent density of 70,000 cells/cm^2^ in NCM and cultured for 24 hours. Cells were washed in CMF-HBSS and then treated with LCM for 16 or 24 hours in the presence of vehicle alone (0.1% DMSO) or 100 µM chloroquine (Fisher Scientific, Loughborough, UK), 250 nM bafilomycin A1, 100 µM leupeptin, 10 µM MG132 (all from Sigma), 20–200 nM bortezomib (a gift from Prof. S. High). Cells were then lysed in DTT sample buffer (100 mM DTT, 10% glycerol, 2% SDS, 80 mM Tris pH 6.8) and analysed by immunoblotting.

### Antibodies

The following antibodies were used for immunoflourescence and western blotting: mouse monoclonal antibodies (mab) against desmoplakin I and II (11-5F) [25], desmoglein 2 (33-3D) [26], desmoglein 3 (32-2B) [27], pan-desmocollin (52-3D) [28], plakoglobin (Sigma) and plakophilin-2 (Progen, Heidelberg, Germany). For immunoflourescence only the following antibodies were used: rabbit polyclonal IgG against γ-tubulin (Abcam, Cambridge, UK), Rab11 (Life Technologies, Paisley, UK) and ninein (a gift from Dr C.Bierkamp), sheep polyclonal IgG against aurora A (a gift from Prof. S.S. Taylor), mouse mab against CLIP170 (Abcam), EB1 (Santa Cruz,) and Lamp1 (a gift from Prof P.G. Woodman). Rabbit antiserum against the cytoplasmic domain of Dsg2 was expressed as a His-tag fusion protein (details to be published at a later date). The following secondary antibodies were used: Alexaflour 488-conjugated goat anti-mouse IgG, Alexaflour 488-conjugated donkey anti-sheep IgG, Alexaflour 488-conjugated rabbit anti-goat IgG (all from Life Technologies), FITC-conjugated donkey anti-rabbit IgG, rhodamine-conjugated donkey anti-rabbit IgG and rhodamine-conjugated donkey anti-mouse IgG (all from Stratech Scientific Ltd., Newmarket, Suffolk, UK). For western blotting only, the following additional antibodies were used: mouse mabs against α-tubulin (Sigma), keratin 8 (LE41) (a gift from Prof. Birgit Lane) and peroxidase conjugated secondary antibodies including goat anti mouse IgG and IgM and goat anti rabbit IgG (Thermo Fisher Scientific, Loughborough, UK).

### Immunoflourescence and image processing

For plasma membrane staining, cells were rinsed in ice-cold PBS and incubated with 40 µg/ml FITC-conjugated concanavalin A (con-A) (Sigma) for 30 minutes at 4°C prior to fixation. Cells were then either fixed in methanol for 10 minutes or in acetone:methanol for 5 minutes at −20°C and incubated with primary antibody for 1–3 hours at room temperature. Cells were rinsed with PBS and incubated with a flourophore conjugated secondary antibody for 30 minutes at room temperature. Cells were then rinsed extensively in PBS, and mounted in Vectashield antifade (Vector Laboratories ltd., Peterborough, UK). Flourescence was assessed either with a Zeiss Axioplan microscope using 63× plan-apochromat oil immersion objective (NA 1.4) an RTE/CCD-1300-Y camera (Princeton Instruments Inc., Trenton, NJ) and Metamorph software (Universal Imaging Corporation, West Chester, PA). Alternatively, for confocal fluorescence microscopy a Nikon Eclipse 90i (Nikon, Surrey, UK) was used to take z-stacks using a 60× oil immersion plan-apo objective (NA 1.4) and images acquired using Nikon EZC1 software. At least 5 random fields per condition were taken. For quantification of DP and γ-tubulin distribution in LCM- treated MDCK, 100–150 cells from several fields were assessed per time point. The degree of colocalisation in whole cell volumes was assessed by using the Image J intensity correlation analysis plugin to calculate the Pearson's correlation coefficient. For centrosomal analysis a threshold was set to restrict analysis to the subcellular region.

### Live-imaging video of Dsc2a-YFP and pericentrin localisation

MDCK cells stably expressing Dsc2a-YFP were seeded at 70,000 cells/cm^2^ in NCM for 24 hours. They were then transiently transfected with 0.4 µg pericentrin-RFP (a gift from Dr Sean Munro) per well of a 24 well plate using 2.4 µL Fugene 6 transfection reagent (Roche Diagnostics Ltd., West Sussex, UK). 24 hours post-transfection, cells were washed extensively with CMF-HBSS and then treated with LCM for 3 hours during which images were taken every 2 minutes on a spinning disc confocal consisting of a Zeiss axio-observer with a 63× plan apo objective (NA 1.4), CSU-X1 spinning disc confocal (Yokagowa), an Evolve EMCCD camera (Photometrics, Tuscon, AZ) and XYpiezoZ stage (ASI, Eugene Oregon) at 37°C and images acquired using Slidebook 5.0 software (3i, Göttingen, Germany).

### siRNA knockdown of desmoplakin

Human desmoplakin siRNA sequences were taken from Wan et al. [29] and are as follows: DP sense AACCCAGACUACAGAAGCAAU, DP antisense AUUGCUUCUGUAGUCUGGGUU (Dharmacon, Chicago, IL). Using Invivogen siRNA wizard v3.1 blast search, scrambled DP sequences were designed and are as follows: scrambled DP sense GCAGAACAACGCAUCAACAUA, scrambled DP antisense UAUGUUGAUGCGUUGUUCUGC (Dharmacon). HaCaT cells were seeded in 6-well plates at 50,000 cells/cm^2^ in NCM for 24 hours. They were then incubated in serum-free DMEM for 2 hours prior to transfection and oligonucleotides transfected at 50 nM using 2 µg/ml Lipofectamine 2000 (Life Technologies) diluted in serum-free DMEM. At 24 hours post-transfection the cells were either treated with LCM for 1 hour and stained for immunoflourescence or lysed in DTT sample buffer and immunoblotted to assess knockdown efficiencies.

### Preparation of MDCK Triton-X100 soluble and insoluble fractions

MDCK II cells were seeded at 90,000 cells/cm^2^ subconfluent density on 90 mm dishes for 24 hours in NCM. Cells were then washed extensively in CMF HBSS and incubated at 37°C in LCM for 90 minutes. Cells were then washed twice in PBS and treated with 0.1% Triton-X100 in PBS containing 1% protease inhibitor cocktail (Sigma) for 20 minutes at room temperature. The Triton-soluble fraction was collected and the remaining insoluble fraction dissolved in DTT sample buffer. DTT sample buffer was added to the Triton-soluble fractions, and insoluble and soluble fractions were equally loaded for protein amount onto polyacrylamide gels, separated by SDS-PAGE and then immunoblotted.

### Desmoglein 2 recycling assay

MDCK cells were seeded at 70,000 cells/cm^2^ subconfluent density in NCM on 60 mm dishes for 24 hours. Cells were then washed extensively with CMF HBSS and either treated with LCM or with a reverse calcium switch (REV) consisting of LCM for 1 hour followed by NCM for 1 hour to stimulate new desmosome formation. At the end of treatment, cells were treated with 0.25% TPCK treated trypsin (Sigma) and 1 mM EDTA (Sigma) for 15 minutes at 37°C. They were then centrifuged and the resultant pellet lysed in boiling DTT sample buffer. Protein was equally loaded on SDS-PAGE gels and immunoblotted.

### SDS-PAGE & western blotting

Protein samples were all prepared in DTT sample buffer, equally loaded at concentrations of 50–100 µg protein per lane and then separated by SDS-PAGE. Protein was then transferred to nitrocellulose membrane, blocked in 5% milk dissolved in PBS/0.05% Tween-20 for 1 hour, then incubated in primary antibody for either 1 hour or overnight. Nitrocellulose membranes were washed, and probed with a secondary HRP -conjugated antibody for 30 minutes. Chemiluminescence (Supersignal West Pico CL kit, Thermo Fisher Scientific) was used to detect bound protein. Where necessary, densitometry was quantified using Adobe Photoshop and normalised with respect to loading control proteins.

### Online supplemental material

Video S1 shows internalised Dsc2a YFP surrounding and then co-localising with the centrosomal marker pericentrin-RFP. Figure S1 shows stills from Video S1 at time points 0 (A), 45 minute (B) and 1 hr 40minutes (C).

## Results

### Half desmosome internalisation is dependent on actin and PKC

We have used the calcium switch model to study desmosome internalisation. This involves chelating extracellular calcium with 3 mM EGTA from the medium surrounding cells in short term culture. Such cells have calcium dependent desmosomes so this process causes loss of desmosomal adhesion generating half desmosomes that are rapidly internalised by the cells [13], [15], [17], [24]. This internalisation of intact plaque-bearing desmosomal structures is essentially similar to the internalisation of whole desmosomes that occurs in vivo and is currently the best culture model of desmosome down-regulation.

**Figure 1:**
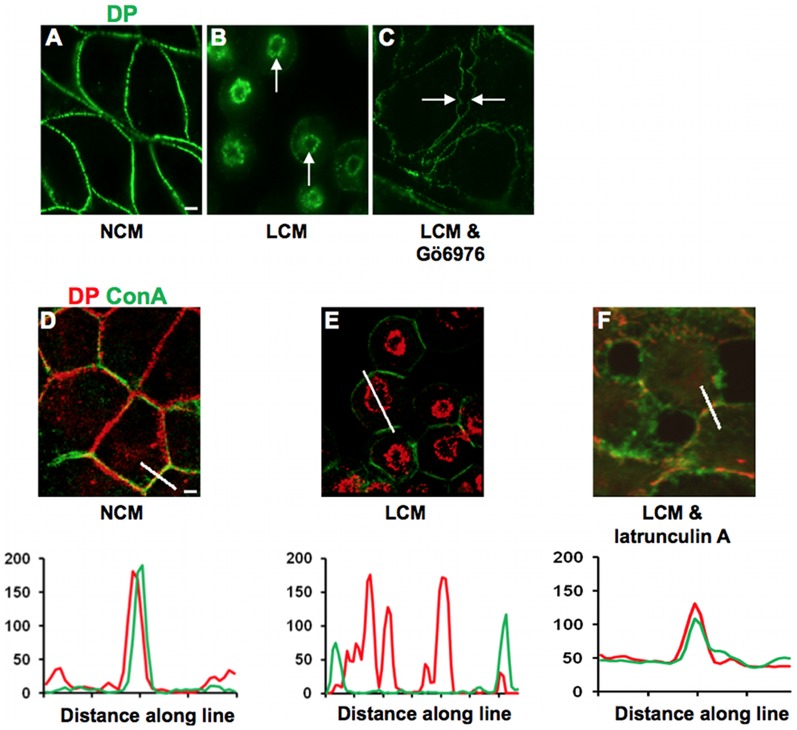
Half desmosome internalisation is cPKC and actin dependent. (A) MDCK cells cultured in NCM had desmosomes at cell-cell contacts as indicated by DP staining. (B) Internalised rings of DP were present in cells treated for 60 minutes with LCM (arrows). (All internalisation controls in LCM were carried out in the presence of the appropriate drug vehicle.) (C) Internalisation of desmosomes was prevented by co-treatment with LCM and Gö6976 (0.8 µM), as cell contact was lost but half desmosomes remained at the cell surface giving rise to the appearance of intercellular gaps (arrows). (D) In NCM, DP (red) was localised to the cell surface in association with the surface marker con-A (green). (E) LCM treatment caused internalisation of DP and separation from con-A, which remained at the surface. (F) Co-treatment with LCM and latrunculin A (5 µM) inhibited desmosome internalisation, as indicated by persistent association of DP with con-A. Fluorescence profiles depict the intensity of staining along the white line in the images.

To test the hypothesis that conventional PKC isoforms are involved in internalisation of half desmosomes MDCK cells were co-treated with LCM and Gö6976 (0.8 µM), an inhibitor of these isoforms. Whilst LCM treatment alone elicited DP internalisation (Figure 1A,B), after co-treatment with Gö6976 the cells detached from each other but DP persisted at the cell surface (Figure 1C) indicating that the desmosomes had split through the extracellular domain but were not internalised. This suggests that conventional PKC isoform activity is required for the internalisation of half desmosomes. In MDCK cells the PKC isoform involved is PKCα [15].

(N. B. Since the effect of PKC signalling on desmosomal adhesion is rapid, this experiment cannot be done by PKCα depletion, because of the time required. Depletion renders desmosomes hyper-adhesive [15]. Such desmosomes do not split on treatment with LCM and are therefore not internalised. In fact we have done the depletion experiment and, as expected, the desmosomes remained intact at the cell surface.)

One of the cellular functions of PKC is regulation of the actin cytoskeleton [30] and both actin and PKC have been implicated in endocytic and phagocytic processes [31], [32], as well as in the internalisation of desmosomes [33]. To confirm the latter result, cells were treated with LCM in the presence of 5 µM latrunculin A to depolymerise actin filaments. (Depolymerisation was confirmed by staining with fluorescent phalloidin (not shown).) This prevented the internalisation of half desmosomes, as demonstrated by the continued co-localisation of DP at the plasma membrane (Pearson's score ∼0.6) (Figure 1F), thus confirming that the actin cytoskeleton is required for internalisation.

### Microtubules and kinesins regulate internal transport of half desmosomes

**Figure 2:**
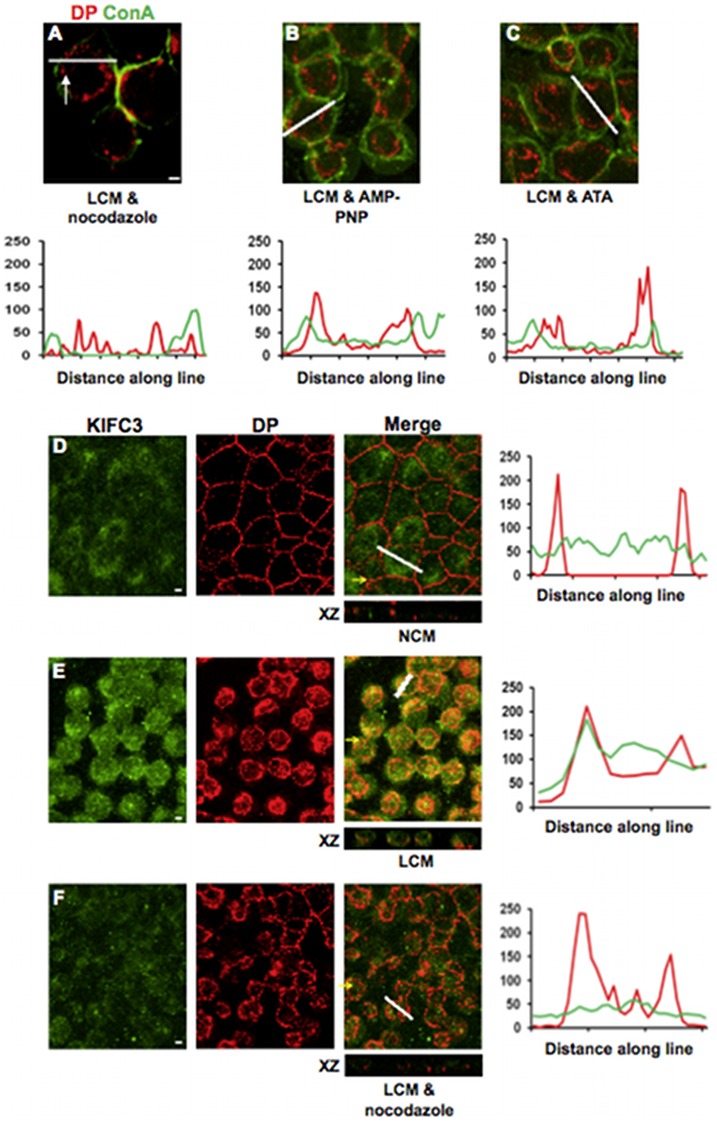
Half desmosome intracellular transport is regulated by microtubules. (A–C) Co-treatment with LCM and nocodazole (33 µM) (A), AMP-PNP (500 µM) (B) or ATA (50 µM) (C) caused DP to be internalised but to remain just beneath the cell surface (arrows). Confocal images of MDCK cultured in NCM and stained for KIFC3 (green) and DP (red) (D) which co-localise following 30minutes LCM treatment (E). This co-localisation was inhibited by LCM co-treatment with nocodazole (33 µM) (F). Yellow arrows indicate XZ axis. Bar, 5 µm. Fluorescence profiles depict the intensity of staining along the white line.

Once internalised, half desmosomes continued to be transported to the perinuclear region of the cells (Figure 1B, E). It is well established that transport of endocytosed proteins and vesicles is carried out by microtubules [34], [35]. To determine whether this is also the case for internalised desmosomal halves, cells cultured in NCM for 24 hours were pre-incubated for 20 minutes with 33 µM nocodazole to depolymerise microtubules and then with LCM in the presence of nocodazole. (Depolymerisation of microtubules was confirmed by immunofluorescence for tubulin (not shown).) In cells thus treated DP was internalised but remained just beneath the cell surface rather than contracting into a tight ring (Figure 2A), suggesting that intracellular transport was impaired. Thus it appears that actin filaments are required for internalisation of half desmosomes but microtubules are responsible for their further transport within the cell.

The kinesins are microtubule motor proteins that bind their cargo to microtubules and are essential intermediaries in microtubular transport [36], [37]. To determine whether kinesins might be involved in the transport of internalised desmosomal proteins MDCKs were pre-treated for 30 minutes with the broad-spectrum kinesin inhibitors adenylylimidodiphosphate (AMP-PNP) (500 µM) or aurintricarboxylic acid (ATA) (50 µM), followed by co-treatment with LCM and the inhibitor. Both inhibitors attenuated the transport of internalised DP, producing distributions that were identical to those elicited by nocodazole-induced microtubule disruption (Figure 2B, C). As the minus end-directed transport kinesin KIFC3 is expressed in MDCK cells [38], double immunoflourescence for DP and KIFC3 was carried out. This showed that whilst KIFC3 staining was diffuse throughout the cytoplasm (Pearson's score ∼0) (Figure 2D), there was co-localisation between KIFC3 and internalised rings of DP following 30 minutes LCM treatment (Pearson's score ∼0.65) (Figure 2E). Furthermore, co-treatment with nocodazole reduced this co-localisation (Pearson's score ∼0.2) (Figure 2F). Thus KIFC3 is a candidate kinesin for transport of internalised desmosomes.

### Half desmosome internalisation does not require IF attachment

**Figure 3:**
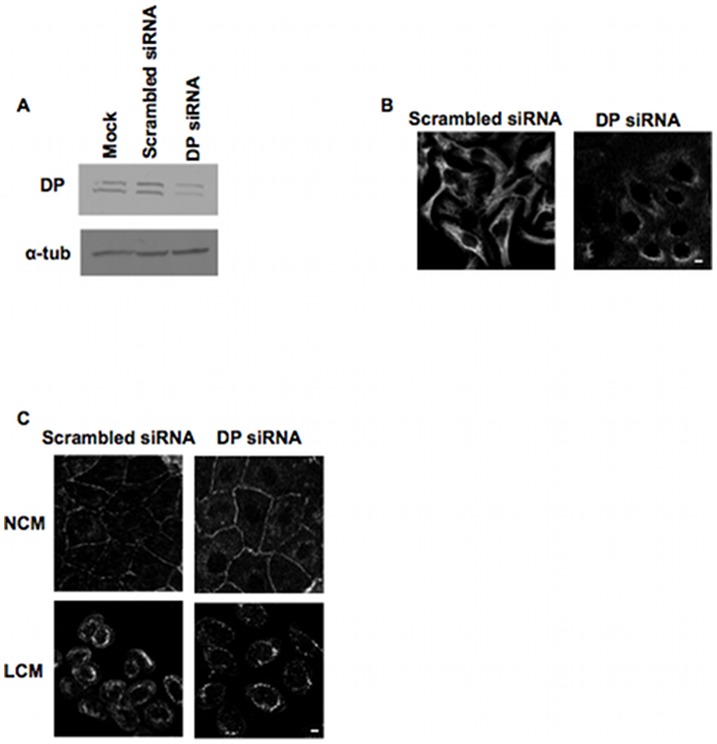
Intermediate filaments are not involved in internalisation of half desmosomes. (A) Western blot showing partial knockdown of DP in HaCaT cells transfected with 50 nM DP siRNA or compared to scrambled DP siRNA and mock transfected controls. (B) Single confocal slices of HaCaT cells or transfected with 50 nM scrambled or DP siRNA showing that the latter cells had disrupted intermediate filament organisation as indicated by keratin 8 & 18 staining. (C) HaCaT cells transfected with DP siRNA or scrambled siRNA were treated with either NCM or LCM for 1 hour and then stained for Dsg2. Half desmosomes were internalised in DP knockdown cells as in the controls. Bar, 5 µm.

Internalisation of half desmosomes appears to depend upon the sequential action of the actin and microtubule cytoskeletons, but DP links the desmosomal plaque to IFs. To determine whether IFs are involved in half desmosome internalisation, we knocked down DP with siRNA, thus disrupting the desmosome-IF link. This experiment was done with HaCaT rather than MDCK cells as the human, but not the canine, sequence for DP is available. Immunoblotting of whole cell lysates showed substantial reduction of DP expression from cells transfected with 50 nM DP siRNA as compared to those transfected with scrambled siRNA or non-transfected samples (Figure 3A). Immunofluorescence for keratin 8 & 18 demonstrated a striking effect of DP knockdown on IF organisation. Cells lacking DP had diffuse, non-filamentous keratin staining that did not link to desmosomes, whilst cells transfected with scrambled siRNA had filamentous keratin (Figure 3B). Immunofluorescence revealed that LCM treatment of cells with DP knockdown were still able to internalise desmosomes; they had distinctive internalised rings of Dsg2 identical to those found in cells transfected with scrambled siRNA (Figure 3C). Thus it appears that the IF cytoskeleton is not required for internalisation of desmosomal proteins.

### Internalised half desmosomes co-localise with the centrosome

**Figure 4:**
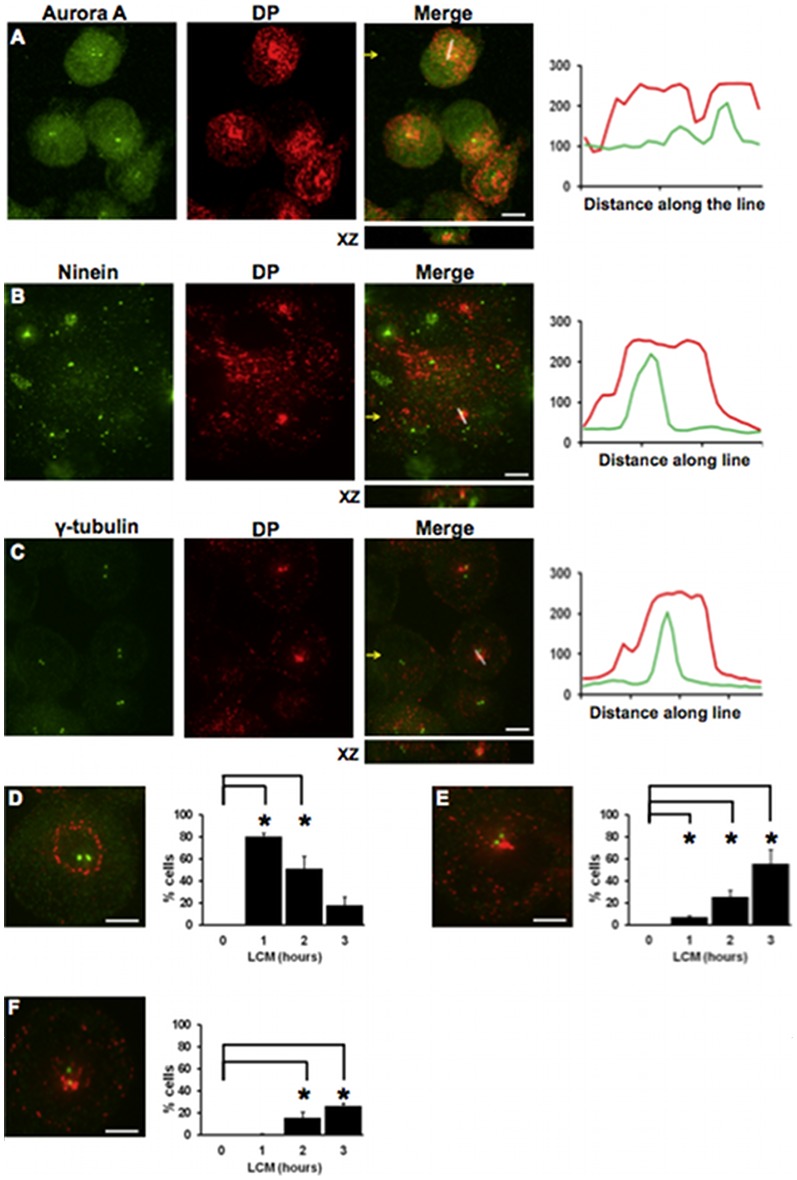
Internalised desmoplakin co-localises with the centrosome. (A–C) Co-localisation of DP with three centrosome markers. Single confocal slices of MDCK cells stained for DP (red) and the centrosome markers aurora A (A) ninein (B) and γ-tubulin (C) (all green) following 2-3 hours of LCM treatment. Fluorescence profiles depict the intensity of staining along the white line in the merged images. (D–F) The time course of co-localisation. Representative images of desmoplakin and γ-tubulin localisation demonstrate the 3 categories of staining pattern used for quantification with DP surrounding the centrosome (D), beside the centrosome (E) or co-localised with the centrosome (F) during LCM treatment, data are mean values ± s.e.m. Yellow arrows indicate XZ axis. Bar, 5 µm. Asterisk indicates statistical significance (p<0.0286, Mann-Whitney test).

Because internalised half desmosomes are transported along microtubules and form a tight ring or dot adjacent to the nucleus, it seemed possible that they may be being transported to the centrosome, the cellular anchor for microtubule minus ends [39], [40]. To test this, MDCK cells were treated with LCM for 2–3 hours to induce internalisation of half desmosomes, double immunofluorescence for DP and the centrosome markers aurora A, ninein or γ-tubulin carried out, and the results assessed by confocal microscopy. Analysis of individual confocal z-slices showed either rings of DP surrounding the centrosome or co-localisation of internalised DP with all 3 centrosome markers (Pearson's score ranged from ∼0.5 to 0.8) (Figure 4A–C). It seemed possible that these alternative distributions represented different stages in the internalisation process. In order to determine whether this was the case, a time course was established by quantifying the distribution patterns at different times after LCM treatment. Since the distribution patterns of DP relative to all three centrosome markers were similar and as aurora A is only present at the centrosome for certain phases of the cell cycle, γ-tubulin staining was used for this study. Confocal z-slices were analysed and cells counted as having one of three staining patterns: (i) a ring of DP surrounding the centrosome; (ii) a condensed spot of DP beside the centrosome; (iii) DP co-localised with the centrosome. After 1 hour of LCM treatment, 80% of cells had a ring of DP surrounding the centrosome (p<0.0286), but by 3 hours this had decreased to just 18% of cells (Figure 4D). However, as LCM treatment time progressed to 3 hours, the number of cells with DP in a dense spot beside the centrosome (Figure 4E) or co-localised with it (Figure 4F) significantly increased from 7% and 1% to 55% and 26% (p<0.0286), respectively. These data indicate that internalised DP is transported towards the centrosome.

To confirm the transport of internalised desmosome proteins to the centrosome, MDCK Dsc2a-YFP cells were transfected with a pericentrin-RFP construct (pericentrin is a centrosome matrix protein and centrosome marker) [41]. Live imaging over 3 hours of cells in LCM showed that the cells initially rounded up and detached from each other (Video S1). By 45 minutes an internalised ring of Dsc2a-YFP was seen surrounding the centrosome (Video S1 & Figure S1B). By 100 minutes, co-localisation of internalised Dsc2a and pericentrin occurred (Video S1 & Figure S1C). These results confirm that internalised desmosomal proteins are transported to the centrosome.

### Internalised half desmosomes do not disassemble

**Figure 5:**
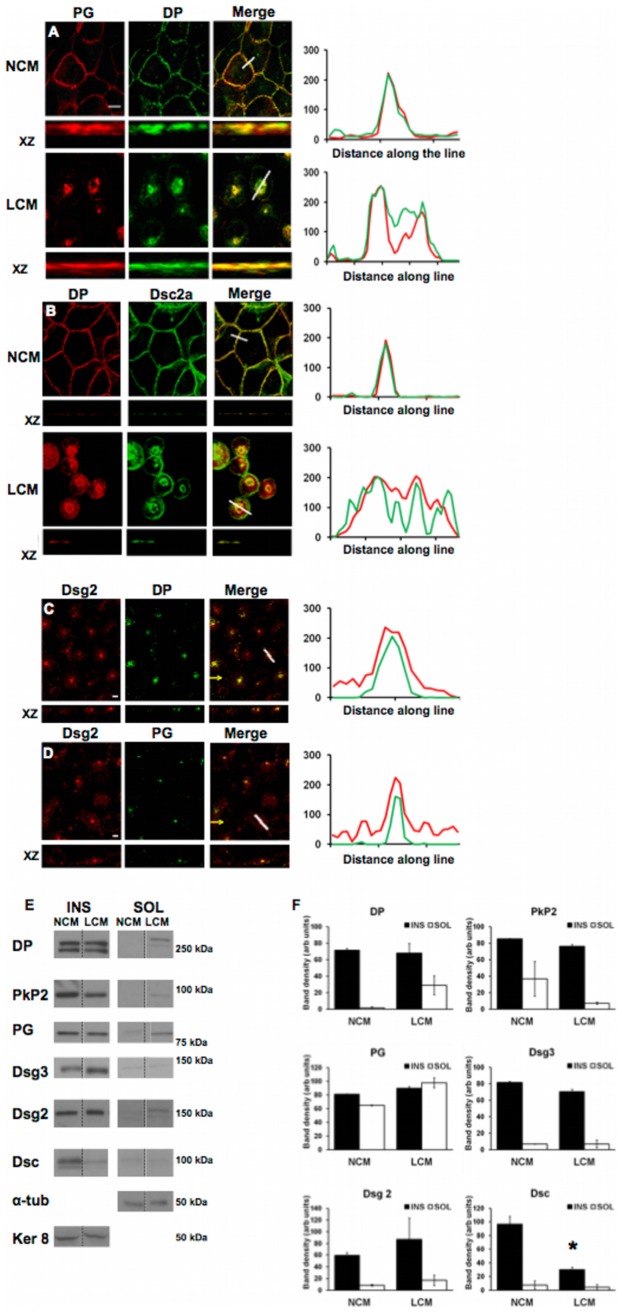
Desmosomal proteins remain co-localised following internalisation. (A–D) Co-localisation of the desmosomal proteins DP, PG, Dsc2a and Dsg2 in NCM persists following LCM-induced internalisation for 1 hour (A,B) and 24 hours (C,D). Yellow arrows indicate XZ axis. Bar, 5 µm. Fluorescence profiles depict the intensity of staining along the white line in the merged images. (E) Cells were treated with either NCM or LCM for 90 minutes and then separated into their insoluble (INS) and soluble (SOL) fractions. Western blots for desmosomal proteins (E) were quantified by densitometry (F) (dashed lines indicate lanes which have been re-ordered from the same western blot). Asterisk indicates statistical significance (p<0.0286, Mann-Whitney test). For further details see text.

To investigate whether internalised half desmosomes disassemble, LCM treated MDCK and MDCK Dsc2a-YFP cells were stained for a range of desmosome proteins. As expected, in NCM the desmosome proteins DP, PG, and Dsc2a were co-localised at points of cell-cell contact (Pearson's score ∼0.9) (Figure 5A, B). Following LCM treatment co-localisation persisted with DP, PG and Dsc2a all continuing to associate with one another in internalised ring-like structures (Pearson's score ranged from ∼0.8 to 0.9) (Figure 5A, B). This co-localisation persisted for up to 24 hours post internalisation (Pearson's score ∼0.7) (Figure 5C, D), suggesting that desmosomal halves remain intact following internalisation.

To support this suggestion, LCM treated MDCK cells were separated into their Triton-X100-soluble and -insoluble fractions. Densitometry of immunoblots revealed that there was little or no change in DP, PKP2, PG, Dsg3 or Dsg2 levels in the insoluble fraction for up to 90 minutes post internalisation (Figure 5E, F). Only the pan-Dsc immunoblot showed a significant reduction of over 60% total protein from the insoluble fraction (p<0.0286). The reduction of Dsc from the insoluble fraction was not accompanied by a concomitant increase of Dsc in the soluble fraction. (We note that there were slight changes in the amounts of DP, PG and PKP2 in the soluble fraction but the significance of these is unclear since they were not accompanied by comparable changes in the insoluble fraction.) These data suggest that desmosomal halves do not disassemble upon internalisation, although there appears to be some loss of Dsc.

### Internalised Dsg2 is not recycled

The recycling of both internalised adherens and tight junction proteins to the cell surface has been well documented [42]–[45]. However, previous work has suggested that this may not be the case for desmosomal proteins since those that are internalised as a result of LCM treatment appear to remain intracellular when new desmosomes are assembled [17], [24]. We attempted to resolve this by biotinylation of cell surface desmosomal cadherins, but the results proved inconsistent, possibly because the molecules in the desmosomal intercellular space were difficult to biotinylate reliably. We therefore devised a method based on the trypsin-sensitivity of cell surface molecules and the trypsin-resistance of intracellular molecules.

**Figure 6:**
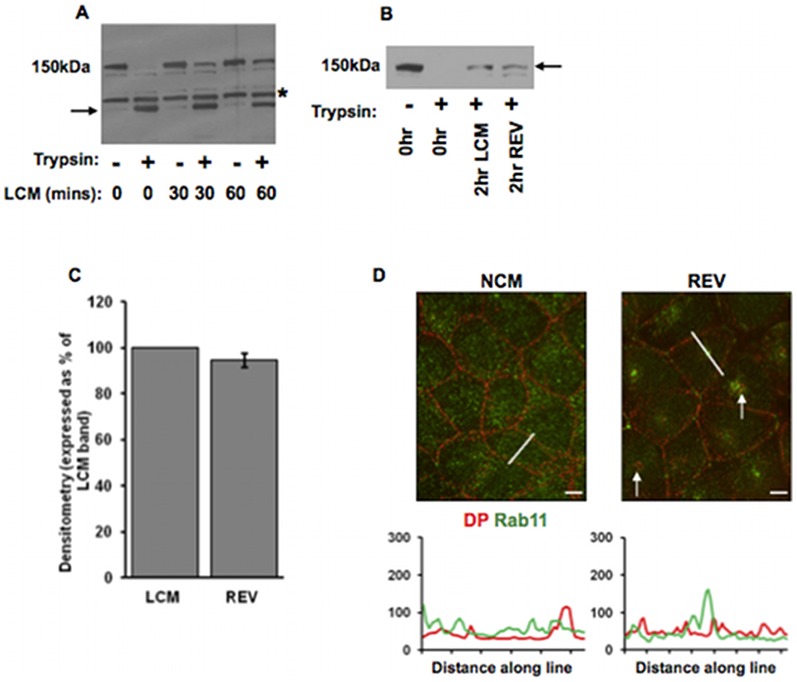
Internalised Dsg2 is not recycled to the cell surface. (A) LCM-induced internalisation protects Dsg2 from trypsin. MDCK cells were treated with trypsin/EDTA after incubation LCM for 0, 30 or 60 minutes. Western blots show that cell surface Dsg2 (150 KDa) was degraded by trypsin/EDTA generating a membrane-protected cytoplasmic fragment (arrow) (LCM (mins) 0). (The band above the trypsin fragment and present in each lane is believed to be a natural degradation product of Dsg2.) However, after LCM treatment a substantial amount of full length Dsg2 remained after trypsin/EDTA treatment (LCM (mins) 30 and 60) showing that it had become membrane-protected. (B, C) The amount of membrane-protected Dsg2 (arrow) remains the same after induction of new desmosome formation following LCM treatment. Cells were treated either with LCM for 2 hours, or with LCM for 1 hour followed by NCM for 1 hour (total time 2 hours) to induce new desmosome formation (verified by immunofluorescence but not shown). The latter treatment is referred to as reverse calcium switching (REV). Western blots (B, quantified in C) show that the amount of membrane protected Dsg2 was identical after both treatments (2 hr LCM, 2 hr REV). Bar in C indicates s.e.m. (D) Internalised DP (arrows) does not colocalise with Rab11 following a reverse calcium switch (REV). Bar, 5 µm. Fluorescence profiles depict the intensity of staining along the white line in the images.

Whole cell lysates of MDCK cells cultured in NCM showed a major band at 150 kDa on western blotting for Dsg2 (Figure 6A). Following whole cell trypsinisation, this band was completely absent, but replaced by a lower band, which represents the membrane-protected cytoplasmic domain of Dsg2 [26] (Figure 6A). However, if the cells were treated with LCM for 30 minutes and then trypsinised, western blotting revealed that some of the full length Dsg2 remained intact, indicating that it had become internalised and hence membrane-protected (Figure 6A). After 60 minutes in LCM, the amount of membrane-protected Dsg2 was increased showing that internalisation was a continuing process (Figure 6A).

To determine whether internalised Dsg2 was recycled to the cell surface, MDCK cells were either treated with LCM for 2 hours alone or for 1 hour, followed by NCM for 1 hour to stimulate new desmosome formation (confirmed by immunofluorescence, not shown. See [17]) followed by trypsinisation to remove residual Dsg2 on the cell surface. There were two possible outcomes of this experiment; either some of the internalised Dsg2 would be recycled to the cell surface in the cells forming new desmosomes which would then be degraded by trypsin, or the internalised Dsg2 would remain internal. In the first case, the density of the internalised full length Dsg2 band should have decreased compared with that in cells that had remained in LCM for the full 2 hours, whereas in the second case, the density of the internalised Dsg2 band should have remained the same in both batches of cells. The result shows that that the densities of the Dsg2 bands were identical (Figure 6B, C). A similar result was observed for Dsg3, with densitometry indicating a 15% reduction of internalised Dsg3 in reverse switch conditions (data not shown). We conclude that the internalised desmosomal cadherins were not recycled to the cell surface. Furthermore, cells treated with a reverse calcium switch were stained for the recycling endosomal marker Rab11 and DP. DP was present both inside cells (Figure 6D, arrows) (caused by the initial LCM treatment) and at the plasma membrane (following subsequent NCM treatment) indicating new desmosome formation (Figure 6D). Internalised DP and Rab11 were not co-localised, substantiating the finding that internalised desmosomal proteins are not subsequently recycled to the plasma membrane (Figure 6D).

### Internalised desmosomal components are degraded by lysosomes and the 26S proteasome

If internalised desmosome proteins are, like Dsg2, not recycled, they must be degraded by the intracellular protein degradation apparatus. Previous reports have shown that internalised desmosomes associate with the late endosomal marker mannose 6-phosphate receptor [46]. Other research has shown association of the ubiquitin activating enzyme E1 with desmosome plaques [47]. However, definitive degradation pathway(s) for internalised desmosome components have not been identified.

**Figure 7:**
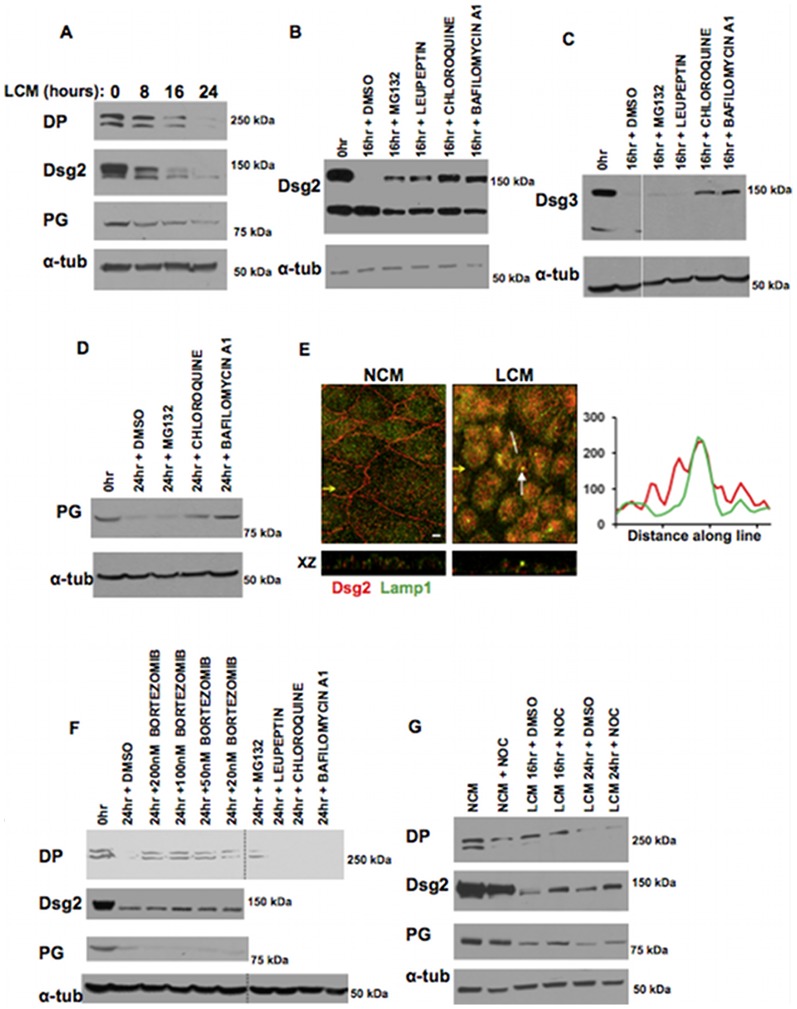
Desmosomal proteins are degraded by lysosomes and proteasomes, and co-localise with the lysosomal marker lamp1. (A) Internalised desmosomal proteins are gradually degraded. Western blots of whole MDCK cell lysates following LCM treatment for 0, 8, 16 or 24 hours show that Dsg2, DP and PG were gradually degraded. (B–D) Lysosomal and proteasomal degradation. Cells were treated with LCM for 16 or 24 hours in the presence of the noted inhibitors or vehicle alone (DMS0 or water). Western blots of whole cell lysates show that the lysosomal inhibitors bafilomycin A1 (250 nM), chloroquine (100 µM) and leupeptin (100 µM) and the proteasomal inhibitor MG132 (10 µM) inhibited degradation of Dsg2 (B) and chloroquine and bafilomycin A1 inhibited LCM-induced Dsg3 and PG degradation (C, D). (E) Internalised Dsg2 co-localises with the lysosomal marker lamp1. Cells cultured in NCM or treated with LCM for 16 hours stained for Dsg2 (red) and Lamp1 (green). Co-localisation in the latter cells is indicated by white arrows.Yellow arrows indicate XZ axis. Bar, 5 µm. Fluorescence profile depicts the intensity of staining along the white line in the image. (F) DP degradation was not inhibited by lysosomal inihibitors, but instead by the proteasomal inhibitors bortezomib (20–200 nM) and MG132 (10 µM) (dashed lines indicate lanes which have been re-ordered from the same western blot). Bortizomib had no effect on PG or Dsg2 degradation (F). (G) Western blots of whole cell lysates co-treated with LCM and nocodazole (33 µM) for 16 or 24 hrs shows degradation of DP is unaltered, whilst degradation of PG and Dsg2 is partially inhibited. Bar, 5 µm.

We first showed that degradation of DP, Dsg2 and PG in LCM-treated cells proceeded gradually (Figure 7A). The rate of degradation varied slightly from one experiment to another but generally amounts had declined to low levels by 24 hours. In order to determine the degradation pathways, cells in LCM were treated with a range of lysosomal and proteasomal inhibitors. The lysosomal inhibitors chloroquine and bafilomycin A1 substantially inhibited the degradation of Dsg2, Dsg3 and PG (Figure 7B–D). Lysosomal degradation of desmosomal proteins was substantiated by co-localisation of Dsg2 with the lysosomal marker Lamp1 in cells treated with LCM for 16 hours (Pearson's score ∼0.7) (Figure 7E).

Intriguingly, no lysosomal inhibitor affected DP degradation. However, the specific 26S proteasome inhibitor bortezomib [48] blocked DP degradation substantially (Figure 7F). Bortezomib had no inhibitory effect on degradation of Dsg2 or PG (Figure 7F). MG132, a non-specific proteasome inhibitor, blocked degradation of both DP and Dsg2 (Figure7, F). These results suggest that (i) internalised desmosomal proteins are differentially degraded by lysosomes and proteasomes, and (ii) the centrosome is not essential in mediating the proteasomal degradation of DP, although disruption of the microtubule network partially disrupts lysosomal degradation.

Proteasomes have been shown to co-localise with the centrosome [49], [50]. To determine whether the co-localisation of internalised desmosomal halves with the centrosome (see above) is essential for desmosome degradation cells were co-treated with nocodazole to block desmosome transport to the centrosome. Nocodazole treatment did not inhibit the degradation of DP, although there was a partial inhibition of PG and Dsg2 degradation (Figure 7G).

## Discussion

**Figure 8:**
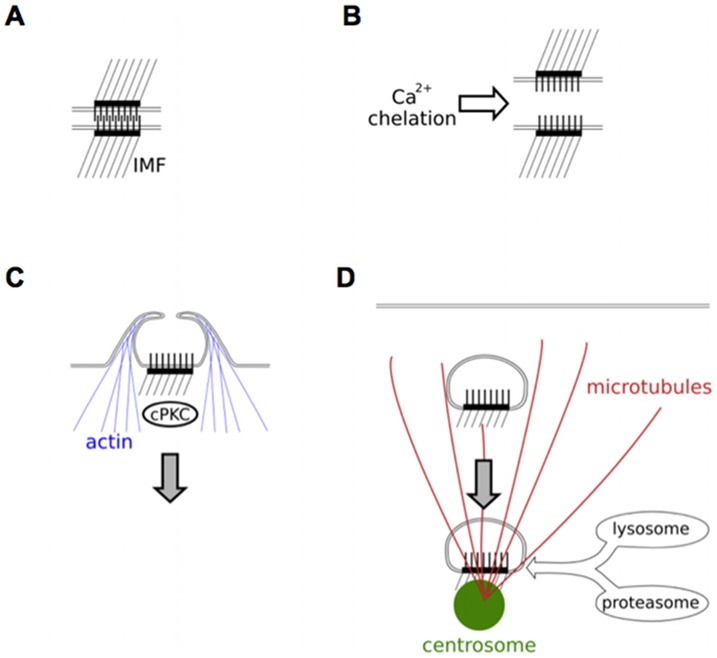
Model of the internalisation, transport and degradation of desmosomal halves. Treatment of calcium dependent desmosomes (A) with LCM causes loss of intercellular adhesion and formation of half desmosomes (B). These are internalised by a mechanism dependent on cPKC and actin filaments (C). Once internalised, desmosomal halves are transported in a microtubule/kinesin dependent manner to the centrosome and degraded by lysosomes and proteasomes (D).

Down-regulation of desmosomal adhesion is required to facilitate epithelial cell migration in embryonic development, wound healing and cancer invasion, but the mechanism of down-regulation is poorly understood. We have provided an outline of the process by which desmosomes are down-regulated in culture following chelation of extracellular calcium (Figure 8). We demonstrate that the internalisation of half desmosomes is dependent on the actin cytoskeleton and conventional PKC isoforms (Figure 8B, C). Once internalised desmosomal halves remain essentially intact and are conveyed to the region of the centrosome by microtubule transport (Figure 8D). Constituent proteins of internalised desmosomal halves are not recycled to the cell surface but instead are slowly degraded by lysosomes and proteasomes. We believe this is the first time that degradation of a cellular organelle has been shown to involve both lysosomal and proteasomal activity.

That the internalised structures remain intact is consistent with our observation that Dsg2 and Dsg3are not recycled; recycling would not be expected unless the desmosomal halves themselves were recycled. Demlehner et al. [51] showed that recycling of desmosomal halves may occur in HaCaT cells, but previous work by us and others suggests that this does not occur in MDCK cells, or at least is not a major mechanism [17], [24]. The process of whole or half desmosome internalisation seems distinct from what is traditionally called “desmosome disassembly”, which implies that desmosomes separate into their component molecules. While our results show that this does not occur in the model we have studied, they do not, of course, rule out the existence of such a process.

Because PKCα becomes associated with desmosomal plaques in wound edge epithelium in association with a weakening of desmosomal adhesion, and because no half desmosomes were detected, we speculated that PKCα might be involved in priming desmosomes for internalisation [12]. If desmosome internalisation is a type of phagocytosis, as postulated by Allen and Potten [11], a role for PKC might be suggested since much evidence suggests that PKC is involved in phagocytosis/endocytosis [52]–[54]. Our observation that inhibition of cPKC isoforms blocks internalisation of desmosomal halves is consistent with a role for PKC, and since PKCα was the only conventional isoform detectable in MDCK cells [15], this is likely to the isoform involved. It will be interesting to discover whether the key targets for PKC are desmosomal proteins or components of the actin cytoskeleton, which is also involved in the internalisation process. It has recently been shown that loss of keratin from the epidermis activates PKCα, causing phosphorylation of DP and destabilisation of desmosomes [55]. Actin, like PKC, plays a key role in phagocytosis [32], [56] providing further support that desmosome internalisation resembles phagocytosis.

We demonstrate that intracellular transport of internalised half desmosomes is microtubule-dependent and probably involves kinesins. Microtubules have previously been associated with two aspects of desmosome function. Firstly, DP is involved in microtubule organisation in differentiated cells of the epidermis [57]. Here desmosomes associate with a number of microtubule end binding/centrosomal proteins including ninein, Lis1, Ndel1 and CLIP170 [58], [59]. We found that neither ninein nor CLIP170 was associated with half desmosomes during the internalisation and intracellular transport processes (not shown). Secondly, microtubules and kinesins have been shown to function in the transport of desmosomal cadherins to the cell periphery during desmosome assembly in cultured cells, although there is some controversy about the involvement of microtubules in desmosome assembly [60]–[62].

Microtubules are organised in a stellate array with their negative ends anchored at the centrosome so that minus end-directed transport should logically end there. Indeed we have shown that internalised half desmosomes first surround, then co-localise with the centrosome. A number of cellular proteins have previously been shown to co-localise with the centrosome. These include IκBα, Dlc-1, hsp70 and p53 [50], [63], [64]. We believe that our work constitutes the first demonstration that an internalised cell surface structure localises to the centrosome.

The significance of localisation to the centrosome may lie in the observation that internalised half desmosomes are degraded rather than recycled to the cell surface. The centrosome has shown to be a site of proteasome localisation [49], [50] where proteasomal activity appears to regulate both centrosome function and the degradation of cellular proteins [49], [50], [65], [66]. We show that DP, the most peripheral desmosomal component, is proteasomally degraded. However, nocodazole-induced blocking of desmosome transport to the centrosome did not inhibit DP degradation possibly indicating shuttling of the 26S proteasome to a differing cellular location as has been well reported [67]. Partial inhibition of Dsg2 and PG degradation by microtubule disruption could be explained by the requirement of intact microtubules for final stages of lysosomal degradation following autophagy [68]. Alternatively, as lysosomes and endosomes are reportedly concentrated close to the microtubule organising centre [69], microtubule disruption may prevent lysosomal degradation there.

Our studies suggest that degradation of internalised half desmosomes is complex, involving both the proteasome and the lysosome. Thus degradation of the desmosomal proteins other than DP was blocked by lysosomal inhibitors and the internalised material co-localised with the lysosomal marker Lamp1. Lysosomal degradation was suggested previously by association of half desmosomes with late endosomes [46], structures which fuse with lysosomes [70].

Our work has revealed a number of novel aspects of desmosome down regulation in this model system. Calcium switching is clearly artificial; extracellular calcium concentrations have to be maintained relatively constant in vivo in order to preserve nerve and muscle function. Nevertheless calcium switching has been widely used in cell biology to study junction assembly. Virtually nothing is known about the mechanism of junction down-regulation in vivo. What little is known suggests that there are certain similarities to the model we have studied. Our overview provides a basis for the detailed mechanistic analysis of each of the steps - internalisation, intracellular transport and degradation - and possibly also for the understanding of the process in vivo. When they are induced to undergo epithelial-to-mesenchymal transition (EMT) by growth factors or artificial serum substitutes, epithelial cells in culture down-regulate desmosomes in the presence of calcium by a process that bears some resemblance to what we describe but which is slower and less synchronous [71], [72].We are now investigating desmosome behaviour during EMT to determine whether it resemble that reveal by calcium chelation.

## Supporting Information

Video S1 **Live-imaging video of MDCK Dsc2a-YFP cells cultured at sub-confluence for 24 hours and then transfected with pericentrin-RFP for a further 24 hours using Fugene 6.** LCM treatment induced detachment and rounding up of cells, and the internalisation of Dsc2a-YFP. Internalised Dsc2a-YFP initially surrounds pericentrin-RFP and is then transported towards it, with colocalisation occurring at around 1 hour 40 minutes LCM treatment. Images were taken every 2 minutes.
(AVI)

Figure S1 **Single images from video S1 show Dsc2aYFP at the plasma membrane at time point 0 (A) surrounding pericentrin-RFP (B) at 45 minutes LCM treatment and co-localising with pericentrin-RFP (C) following 1 hour 40 minutes LCM treatment.**
(TIF)
