# Supplementary figures and images for: Down-Regulation of Desmosomes in Cultured Cells: The Roles of PKC, Microtubules and Lysosomal/Proteasomal Degradation
Source: PLoS One. 2014 Oct 7;9(10):e108570. doi: 10.1371/journal.pone.0108570 (PMC4188543; doi:10.1371/journal.pone.0108570)

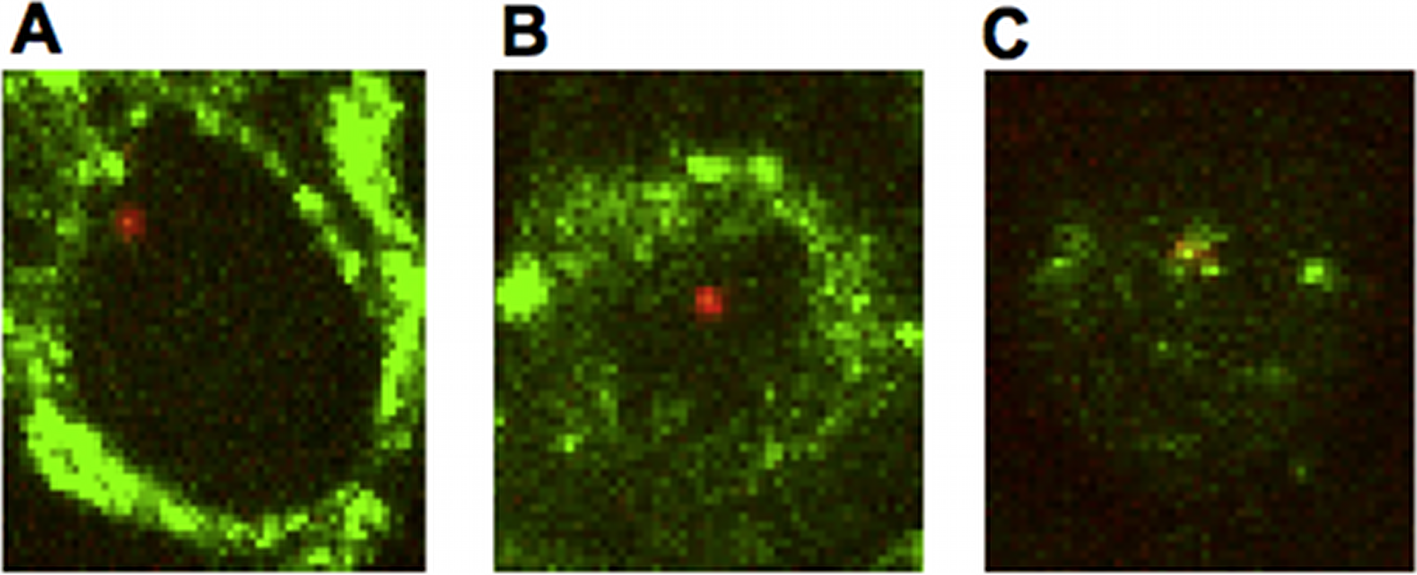

Supplement: Figure S1 — Single images from video S1 show Dsc2aYFP at the plasma membrane at time point 0 (A) surrounding pericentrin-RFP (B) at 45 minutes LCM treatment and co-localising with pericentrin-RFP (C) following 1 hour 40 minutes LCM treatment. (TIF) [file pone.0108570.s002.tif]
